# Ultrafast decoherence dynamics govern photocarrier generation efficiencies in polymer solar cells

**DOI:** 10.1038/srep29437

**Published:** 2016-07-14

**Authors:** Eleonora Vella, Hao Li, Pascal Grégoire, Sachetan M. Tuladhar, Michelle S. Vezie, Sheridan Few, Claudia M. Bazán, Jenny Nelson, Carlos Silva-Acuña, Eric R. Bittner

**Affiliations:** 1 Department of Physics and Regroupement québécois sur les matériaux de pointe, Université de Montréal, C.P. 6128, Succursale centre-ville, Montréal H3C 3J7, Canada; 2 Department of Chemistry, University of Houston, Houston, Texas 77204, USA; 3 Department of Physics, Blackett Laboratory, Imperial College London, London SW7 2AZ, United Kingdom

## Abstract

All-organic-based photovoltaic solar cells have attracted considerable attention because of their low-cost processing and short energy payback time. In such systems the primary dissociation of an optical excitation into a pair of photocarriers has been recently shown to be extremely rapid and efficient, but the physical reason for this remains unclear. Here, two-dimensional photocurrent excitation spectroscopy, a novel non-linear optical spectroscopy, is used to probe the ultrafast coherent decay of photoexcitations into charge-producing states in a polymer:fullerene based solar cell. The two-dimensional photocurrent spectra are interpreted by introducing a theoretical model for the description of the coupling of the electronic states of the system to an external environment and to the applied laser fields. The experimental data show no cross-peaks in the twodimensional photocurrent spectra, as predicted by the model for coherence times between the exciton and the photocurrent producing states of 20 fs or less.

Advances in the fabrication of organic polymer-based photovoltaic (OPV) cells have led to power conversion efficiencies (PCE) in excess of 10% in certain polymer:fullerene devices, making them viable materials for light-harvesting devices^1^. However, the fundamental photophysical pathways that connect the absorption of light to the production of charge carriers remain elusive in spite of rigorous experimental and theoretical investigation. OPVs based on the so-called bulk heterojunction consist of blends of electron donor (usually a *π*-conjugated polymer) and electron acceptor (typically a fullerene derivative or a second polymer) materials.

The bottleneck that has attracted the attention of theorists and experimentalists alike is that the primary photoexcitations are tightly bound intramolecular excitons with high-binding energy (≈500 meV). Subsequently, the lowest-energy intermolecular charge-transfer (CT) states that occur at the phase-boundary between donor and acceptor materials also have binding energies in the range of 300–400 meV^2^. The general paradigm for some time has been that the CT states are the primary precursors to photocarriers. However, in order to produce photocurrent, the electrons and holes produced by photoexcitation must separate far enough such that their binding energy could be small as compared to the thermal energy near ambient conditions.

Recent spectroscopic measurements on organic photovoltaic systems report that charged photoexcitations can be generated on ≤100-fs timescales^3^,^4^,^5^,^6^,^7^,^8^,^9^,^10^,^11^. Delocalization of the transferred electron amongst several fullerenes diminishes its Coulombic attraction to a hole localized in the polymer (donor) phase. Transient absorption experiments by Gélinas *et al*., in which Stark-effect signatures in transient absorption spectra were analysed to probe the local electric field as charge separation proceeds, indicate that electrons and holes separate by ≈40 Å over the first 100 fs and evolve further on picosecond timescales to produce unbound charge pairs^12^. Model calculations based upon Fermi’s golden rule also indicate that such rapid time-scales are only consistent with a model of a highly-delocalised charge in the acceptor phase^12^. Concurrently, Provencher *et al*. demonstrated, via femtosecond stimluated Raman spectroscopic measurements, the emergence of clear polaronic vibrational signatures on sub-100-fs on the polymer backbone, with very limited molecular reorganization or vibrational relaxation following the ultrafast step^13^. Such spectacularly and apparently universally rapid through-space charge transfer between excitons on the polymer backbone and acceptors across the heterojunction would be difficult to rationalize within Marcus theory using a localised basis without invoking unphysical distance dependence of tunnelling rate constants^14^. In addition, intramolecular electron-vibration interactions in organic conjugated polymers are known to be relatively strong and do contribute to the photophysical dynamics. Recent observations of quantum beating with a time-period of 23 fs in a prototypical polymer:fullerene OPV system suggest that C=C stretching modes of the polymer and the pinching mode of the fullerene are as important as the off-diagonal electronic transfer integral itself^15^, a scenario not unlike what occurs in photosynthetic light-harvesting systems^16^. Similarly, Song *et al*. demonstrated, by means of two-dimensional coherent spectroscopy, the role that such vibrational coherence plays in ultrafast charge generation processes in polymer:fullerene systems^17^. Such observations underscore the importance of coherent vibronic coupling between electronic and nuclear degrees of freedom in charge delocalization and transfer in a noncovalently bound reference system^18^.

We recently proposed that quantum mechanical tunnelling processes brought about by environmental fluctuations couple photoexcitations *directly* to photocurrent producing charge-transfer states on a sub 100-fs timescales^19^. Our heuristic model is supported by a recent numerically exact study of a model spin-boson system with separated diagonal and off-diagonal baths by Yao *et al*.^20^. This work indicates that there exists a critical parametric regime that produces neither localized nor delocalized states and is free of quantum decoherence, suggesting that the existence of such decoherence-free subspaces is critical to the formation of quantum coherent exchange between the exciton and the delocalised charge-separated (CS) states^20^,^21^.

In the present work, we exploit the superior detection sensitivity of ultrafast photocurrent probes^22^ to explore photocarrier generation dynamics by means of two-dimensional (2D) photocurrent excitation spectroscopy (2DPCE). This is a novel nonlinear optical spectroscopy in which the two-dimensional excitation correlation spectrum is measured as a function of time after initial excitation with a femtosecond pulse sequence via photocurrent^23^,^24^. In general, 2D electronic spectroscopies permit identification of homogeneous and inhomogeneous spectral lineshape contributions, and because they are nonlinear optical techniques, they can reveal the existence of couplings between distinct excited states through the presence of cross peaks (off-diagonal signals) in the 2D correlation spectrum. Because of these unique advantages over linear techniques, 2DPCE is extremely valuable in the investigation of the photocharge generation dynamics in materials in which photocarriers are not produced directly by inter-band transitions as in bulk semiconductors, but in which precursors to photocarrier pairs, such as excitons in molecular semiconductors, are the primary photoexcitations. We use 2DPCE in order to explore the ultrafast decoherence dynamics between mixed exciton and delocalised polaron states that we proposed to be photocarrier precursors in ref. 19. We begin with a brief overview of a theoretical model describing salient electronic states of this system and their coupling to an external environment and to the applied laser fields. This will serve as an important touchstone in interpreting our experimental data. We then describe the results of our 2DPCE experiments on a working organic polymer based solar cell based upon a polymer:fullerene blend.

## Results

### Theoretical model

**Figure 1:**
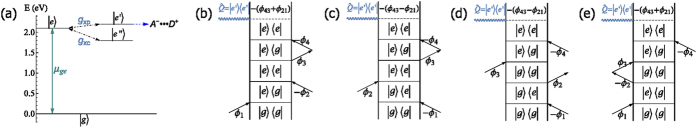
(**a**) Schematic of the relevant energy levels in our model along with their couplings. (**b**–**e**) Double-sided Feynman diagrams contributing to the 4th order population. Incoming and outgoing arrows indicate interactions with the laser fields along with their phase. 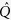 is the final projection onto the outgoing polaron states that contribute to the photocurrent.

We begin by developing a model for the salient electronic states of an organic heterojunction system as sketched in Fig. 1a, in which we assume that the primary photoexcitation (exciton) is produced by the absorption of a photon from the electronic ground state of the system and can decay into either a charge-transfer (CT) state pinned to the heterojunction interface, or into delocalised charge-separated (CS), or polaron pair, states^19^. We also assume that population in the CS states is ultimately responsible for any photocurrent produced by the actual device. Consequently, in the limit of no other channels for carrier loss, photocurrent is at least proportional to the net population transferred to the CS states. Moreover, both spectroscopic measurements and quantum chemical studies place the CT state energetically well below the threshold for polaron formation such that these CT states cannot decay into CS states via thermally activated processes^2^.

We discuss here the results of a series of quantum chemical calculations we performed to verify and support the various parameters used in the phenomenological 3-state model used for computing the 2D spectra. To model any particular physical system, we need information about the energy and nature of the excited states at the donor:acceptor interface where charge separation occurs. The challenge of calculating the properties of such states is complicated by the wide variety of interfacial molecular packing arrangements and by the size of the interfacial region. This remains an active area of research^25^. In the present case, we use the simplest possible model of the donor:acceptor interface, a complex consisting of an oligomer of the donor polymer and a single fullerene derivative, separated by an edge to edge distance of 3.5 Angstrom, and calculate the lowest excited states in vacuum using time-dependent density functional theory (TD-DFT) with the hybrid B3LYP/6–31G* functional. We classify the resulting states as excitonic, charge-transfer (CT) or mixed according to what fraction of an electron charge lies on the fullerene, and we study the dependence of the energy and type of these states on the relative position of oligomer and fullerene molecule. Our calculation method is described in detail in ref. 26.

For a donor:acceptor pair where the molecules’ HOMO and LUMO energies are staggered, such calculations typically reveal at least one CT state lying at an energy below the lowest exciton state of either donor or acceptor. This qualitative finding agrees with experiment, where optically absorbing charge transfer states have been observed in many polymer: fullerene combinations using high sensitivity absorption^27^,^28^, photocurrent spectroscopy^29^, photoluminescence^30^,^31^ and electroluminescence^32^. The TDDFT approach, although simple, has worked remarkably well in reproducing the observed CT state behaviour; it was shown previously by Few *et al*. to reproduce the observed trend in lowest excitation energies (determined through electroluminescence) with the chemical structure of donor and acceptor constituent materials. In a detailed study, TD-DFT with B3LYP was found to agree with experimental data as well as the more expensive GW many body theory^33^. We acknowledge that TD-DFT will have limitations, and that other approaches to CT state calculation are being developed using higher levels of theory on molecular pairs^34^,^35^, and with a more detailed description of the role of surrounding molecules in determining energetics of interfacial states through polarisation^36^,^37^ and electronic delocalisation^38^,^39^,^40^,^41^. However, for the purpose of the present paper, the method is used only to indicate the type of CT states that will be present in our model system.

Here, we focus on the donor: acceptor system that we study experimentally, the polymer poly(N-9″-hepta-decanyl-2,7-carbazole-alt-5,5-(4′,7′-di-2-thienyl-2′,1′,3′-benzothiadiazole)) (PCDTBT) combined with the fullerene derivative phenyl C-61 butyric acid methyl ester (PCBM). We model the polymer as a trimer in its optimised geometry and study the excited state spectrum for different relative positions of fullerene and oligomer. For each fullerene alignment, we calculate three CT states (more then 0.9 e transferred from oligomer to fullerene) at excitation energies below that of the first oligomer singlet, and a number of “mixed states” (between 0.1 e and 0.9 e transferred from oligomer to fullerene) at energies close to that of oligomer singlet transitions. When the PCBM molecule is aligned with the benzothiadiazole (BT) unit of the oligomer, we calculate an additional CT state with an excitation energy ≈150 meV above that of the oligomer singlet. For each CT state we calculate the Coulomb interaction due to partial charges on each molecule, *U*_*B*_, which gives an indication of the energy required to generate a free-charge pair. (Note that dielectric screening is not taken into account when calculating this value, and it should be used as a guide to relative binding of states, rather than as an absolute value.)

**Figure 2:**
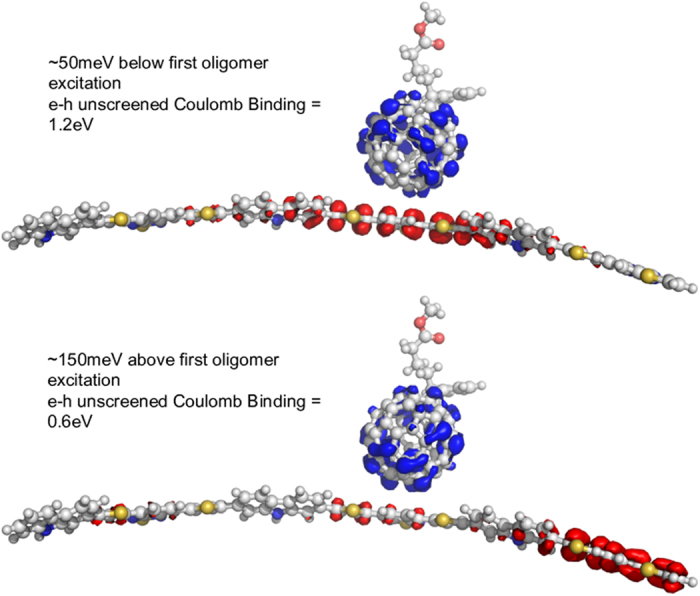
Redistribution of charge, excitation energy, and unscreened Coulomb binding energy for two excitations of a CDTBT oligomer: PCBM molecule pair. Isosurfaces show change in charge distribution between ground state and excited states (electron density moves from red to blue regions). We stress that these are just exemplary structures showing one possible configuration of the pair, and that the states shown are only two out of the ensemble, selected to show the typical energy and nature (degree of CT) of excited state of the donor:acceptor complex. They are not exhaustive.

**Figure 3:**
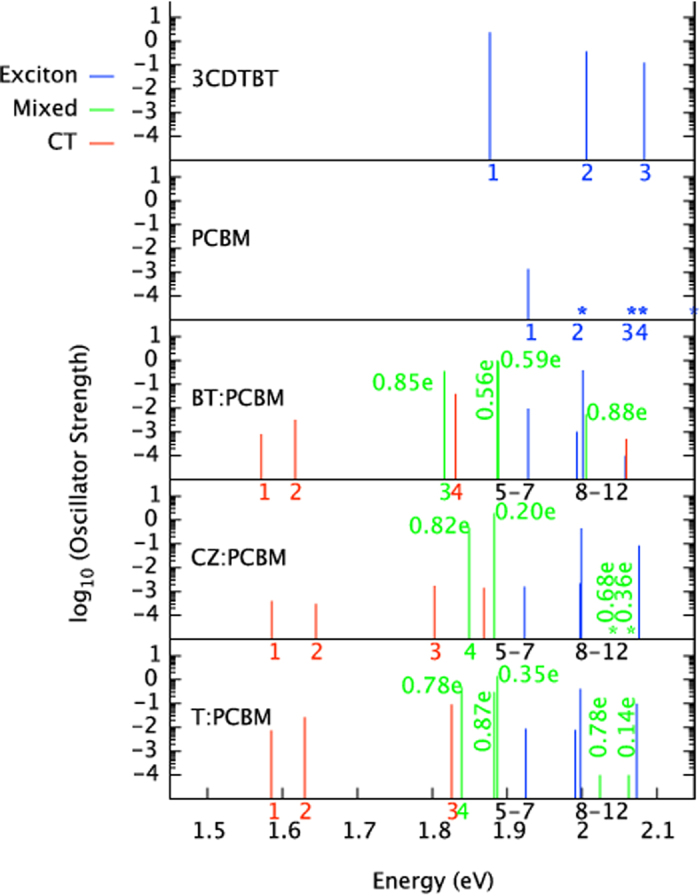
Calculated excitation spectrum for a 3CDTBT oligomer and a PCBM molecule, each alone, and as a molecule pair. Excitations are coloured according to degree of charge transfer exhibited. Single molecule excitations (CT ≤ 0.10 e) are blue, complete charge transfer excitations (CT ≥ 0.90 e) are red, and mixed excitations (0.10 e < CT < 0.90 e) are green.

Figure 2 illustrates the charge redistribution for two of the possible excitations of an oligomer:fullerene pair in the configuration when the fullerene is located 3.5 Å above the central benzothiadiazole unit. In this configuration, we calculate an additional CT state with an excitation energy ≈150 meV above that of the oligomer singlet as well as a CT state that lies 50 meV below. The two states have a different degree of charge transfer character and Coulomb binding between electron and hole. We also find that the higher energy CT state has a lower unscreened electron/hole binding (*U*_*B*_ ≈ 0.6 eV) than the lower energy CT states (*U*_*B*_ ≈ 1.2 eV). A similar trend in decreasing Coulombic binding energy with increasing CT state energy has been reported in a variety of other donor: acceptor blends^26^,^42^. Figure 3 shows the spectrum of excited states for the donor trimer (3CDTBT), the PCBM molecule, and the 3CDTBT:PCBM molecule pair with the fullerene located above the benzothiadiazole unit (BT), the carbazole unit (CZ) and the thiophene unit (T), calculated as described in ref. 26. The configuration of oligomer and fullerene were chosen to be representative of just one of the arrangements likely to be found in the solid film. The resulting states are representative of those likely to be found for this material system, but not exclusive. Alternative approaches^43^,^44^ may lead to different optimised pair conformations and state energies, but these variations will still be minor compared to the variations expected due to disorder in the film, and to variation in the position of the fullerene relative to the polymer backbone (as shown in Fig. 3).

The quantum chemical results show clearly that the excitonic state is bracketed by a intermolecular charge-transfer state lying ≈50 meV below with an unscreened electron/hole binding energy of 1.2 eV and an intermolecular charge-separated state lying at 150 meV above with a unscreened electron/hole binding energy of 0.6 eV. It should be pointed out that molecular calculations such as these do not take into account neighboring molecular units that would otherwise be present in the physical system. The presence of neighboring molecules allows the electron and hole to further delocalise over multiple units thereby both weakening the net Coulombic interaction between the electron and hole and providing an increased density of charge-separated states that the exciton can decay into.

To cast our quantum-dynamical model involving excitations in Fig. 1a into a succinct theoretical framework, we consider the time-evolution of the electronic states of the model system as an open quantum system under influence of a series of co-linear phase-modulated laser pulses. In our calculations, an operator *A*(*t*) corresponding to arbitrary quantum observable can be propagated according to the Lindblad master equation


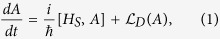


in which *H*_*S*_ is the unperturbed Hamiltonian of the electronic system in its second quantization form


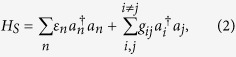


where 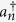 and *a*_*n*_ are fermion operators that create and annihilate excitation quanta in state *n*, and *ε*_*n*_ and *g*_*ij*_ are site energies and tunnelling constants, respectively. The energies and couplings are chosen to correspond to the relevant states in the physical system. We explicitly include the electronic ground state |*g*〉, which is coupled via the transition dipole *μ*_*ge*_ to a single excitonic state, |*e*〉, which in turn is coupled to a dark charge transfer state |*ct*〉 with electronic coupling *g*_xc_, and a single charge separated (polaron) state |*p*〉 with coupling *g*_xp_. We assume that photocurrent arises from the decay of population in state |*p*〉 into a continuum of bulk polaron states on a timescale much longer than any other timescale in our system. Figure 1a gives schematic of the relative placement of the various states explicitly considered in our model. We also assume that the internal electronic dynamics are coupled to a dissipative environment that modulates the electronic energies as well as electronic transfer integrals *g*_xc_ and *g*_xp_. The Lindblad terms in the 3-state model provide a succinct avenue for incorporating the local vibronic environment–including effects due to nearby molecular units–directly into the light-matter quantum dynamics that give rise to the 2DPCE spectra.

We introduce the interactions with the external environment using the Lindblad approach, following a suitable set of assumptions including that at the initial time, the system and environment are uncorrelated and that the environment is large enough so that its memory time is very short. This latter assumption is critical since it allows the use of a Markovian approach. The Lindblad approach is commonly used in quantum optics where both of these assumptions are easily met. For the case at hand, in which the environment consists of the electronic polarizibility and vibrational (phonon) response of the molecular surroundings, the Markov assumption may be difficult to justify but nonetheless provides a useful starting point for describing the dynamics of our system. Using Lindblad master equation Eq. 1, the time evolution of an operator in the system subspace can be determined with the Lindblad dissipation term 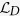 given by


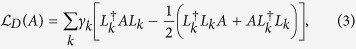


where *γ*_*k*_ are the relaxation amplitudes and *L*_*k*_ are the Lindblad operators that characterize the effect of the environment on the system. Decoherence and relaxation effects on the electronic degrees of freedom can be attributed to the oscillation of energy levels and the modulation of the transfer integral via Lindblad operators 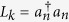 and 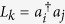 (*i*≠*j*). Primary analysis^45^,^46^ based on a two-level system indicates that population relaxation time scale *τ*^−1^ = *γ* is merely dominated by the environment facilitating state transition in the Hamiltonian (the so-called off-diagonal coupling), whereas the decoherence process is determined by the bath modulation on both energy levels and tunnelling terms, with the former being referred to the mechanism of diagonal coupling. Using this approach, we can effectively model both relaxation and decoherence within the context of a single theoretical framework, taking the system-environment couplings as phenomenological variables. For example, the timescale for decoherence between two electronic states is ultimately set by the fluctuations in the couplings between the states *viz*.


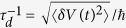


where *δV*(*t*) is a random variable with 〈*δV*(*t*)〉 = 0 stemming from environmental fluctuations (*e*.*g*. phonons) described by a single spectral density *S*(*ω*) of the environment.

In the 2DPCE experiment, it is natural to assume that the total photocurrent signal is proportional to the population of the polaron state |*p*〉


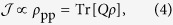


in which *Q* = |*p*〉 〈*p*| is the projection operator that we take to be the precursor for any photocurrent produced by the pulse sequence. We take the light-matter coupling as a weak perturbation and expand the time-dependent density operator in the powers of the applied laser field as


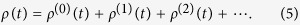


The polaron population to the *n*th order can be evaluated as 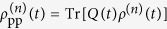, i.e.,


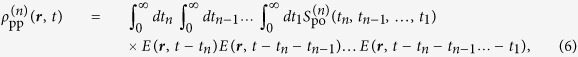


where *E*(***r***, *t*) is the electric field component of the lasers. Note that the time-ordered expansion is in terms of the intervals between field-matter interactions denoted by 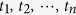, with *t*_1_ being the earliest and *t*_*n*_ being the last. The *n*th order polaron population susceptibility is


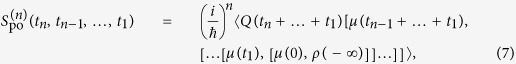


where *μ*(*t*) is the electronic dipole operator evolved in time using the Lindblad master equation described above. Comparing this to conventional methods for nonlinear spectroscopy, such as a four-wave-mixing experiment ending in the ground state, the outgoing signal of this photocurrent spectroscopy is governed by the polaron population^47^. It is also important to note that only the even order susceptibilities are nontrivial according to the electric field symmetry. We compute the fourth order susceptibility of the polaron population which is the lowest order to obtain 2D spectrum. Consequently, the simplest nonlinear photocurrent spectroscopy corresponds to the 4th order susceptibility technically involving 8 correlation functions instead of four in the 3rd order spectroscopy. Regarding the scheme of phase-modulation detection^47^, a detailed analysis of the susceptibility based our model indicates four survived contributions that can be categorized as rephasing *ϕ*_*RP*_ = *ϕ*_4_ − *ϕ*_3_ − *ϕ*_2_ + *ϕ*_1_ and non-rephasing *ϕ*_*NRP*_ = *ϕ*_4_ − *ϕ*_3_ + *ϕ*_2_ − *ϕ*_1_ signals, with respect to the phases of the four collinear laser pulses, as depicted by the double-side Feynman diagrams in Fig. 1, corresponding to two stimulated emission (Fig. 1(b,c)) and two ground state bleach (Fig. 1(d,e)) pathways. The rephasing signal in the phase-modulation 2DPCE spectroscopy is analogous to the photon echo spectroscopy under the four-wave mixing scheme. Note that excited state absorption are ruled out in such single excitation manifold. The final signal results from interference between each of these possible pathways. If, in fact, there is a direct quantum mechanical link between the initial excitation and any photocurrent precursor state, such links should be revealed by a cross-peak in the 2DPCE signal. However, whether or not such cross-peaks can be resolved depends upon the rate of decoherence between the exciton and the precursor state.

It is worth underlining that this theoretical model for the evaluation of the 2DPCE spectra does not include post-excitation processes. This circumstance is anyway reasonable in the frame of our experimental settings. As it will be described in more detail in the following section, in this work all the 2D spectra were measured under a ≈0.7 V bias, which is the optimal operating condition for the photo cell.

**Figure 4:**
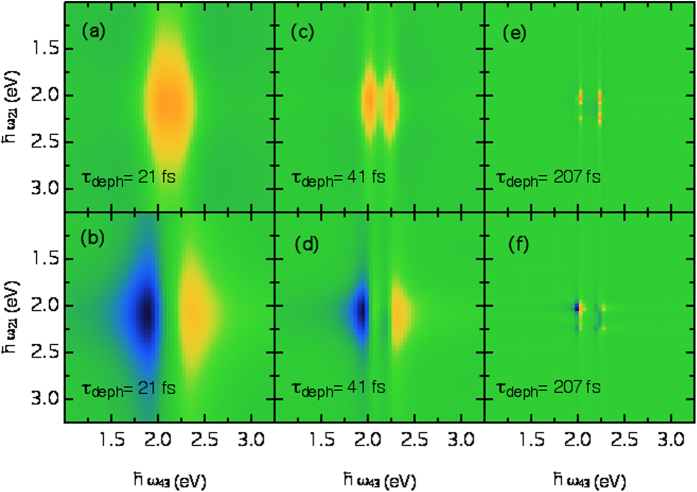
Theoretical 2DPCE total correlation functions for the model system with increasing decoherence times (*τ*_*d*_ = *ħ*/*γ*). The top row shows the real part of the response, while the bottom row shows the imaginary part.

In Fig. 4 we present the results of our quantum simulations of the 2DPCE signals produced by our model parametrized to describe the PCDTBT:PCBM system. We take the decoherence time as a free parameter and compare results at increasing coherence times between the exciton and the polaron states. If *τ*_*d*_ = 21 fs, the 2DPCE response is featureless (Fig. 4(a,b)). As the coherence time increases, one should expect to see that cross-peaks begin to emerge between the exciton and the polaron states (here at 2.3 eV). Indeed, taking this 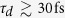 reveals such cross-peaks (Fig. 4(c,d)). In fact, when the decoherence time is taken to be very large (Fig. 4(e,f)), the individual diagonal and off-diagonal spectral components become very well resolved.

### 2D photocurrent excitation spectroscopy

**Figure 5:**
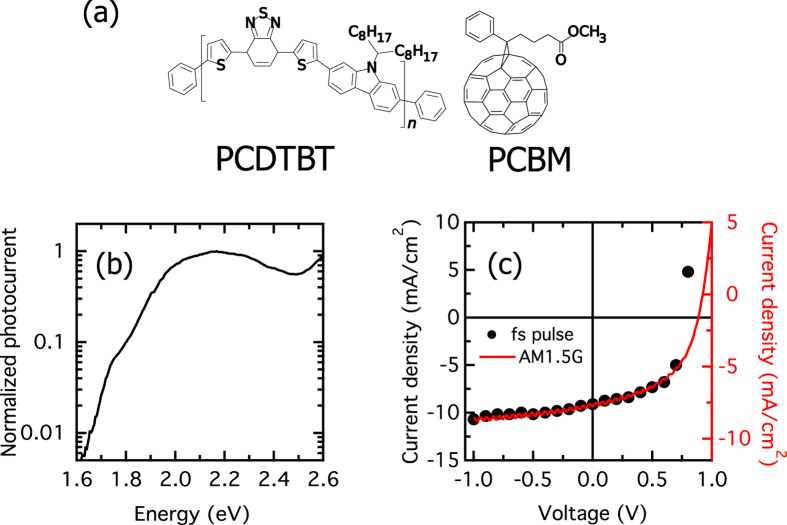
(**a**) Structure of PCDTBT (left) and PCBM (right). (**b**) Photocurrent excitation spectrum of the PCDTBT:PCBM solar cell under investigation. (**c**) Current-voltage response of same cell under AM1.5 G solar illumination (right-axis) and under ultrafast laser illumination (left-axis). The uncertainty in the photocurrent density measured by exciting with the femtosecond pulse train is less than 10%.

**Figure 6:**
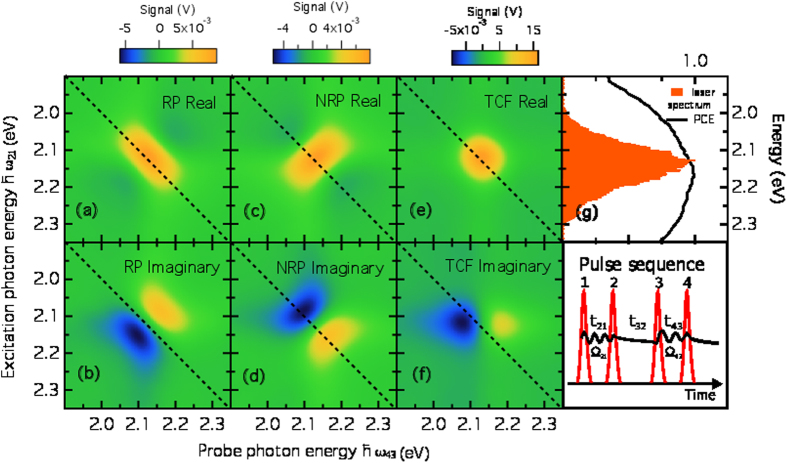
Two dimensional coherent photocurrent excitation (2DPCE) spectra at a population waiting time *t*_32_ = 0 fs measured under a bias voltage of ≈0.7 V. (**a**,**b**) Real and imaginary components of the rephasing (RP) signal. (**c**,**d**) Real and imaginary components of the non-rephasing (NRP) signal. (**e**,**f**) Real and imaginary components of the total correlation function (TCF). (**g**) Laser spectrum and photocurrent excitation spectra over the same range and schematic of the experimental pulse sequence.

We carried out 2DPCE spectroscopy on a solar cell based on PCDTBT:PCBM blend. The molecular structures of PCDTBT and PCBM are shown on Fig. 5(a) and the device structure is described in the Methods section. This is a benchmark polymer:fullerene system which has been shown to yield solar-power conversion efficiencies as high as 7%^48^,^49^. Figure 5(b) presents the linear photocurrent excitation spectrum of such solar cell. 2DPCE measurements were performed exciting the solar cell with a sequence of four collinear ultrafast laser pulses. Figure 5(c) reports the current density as a function of external bias (JV curve) of the cell under both femtosecond-laser and solar (AM1.5 G) illumination. The agreement between the JV curve measured under fs-pulse excitation and the one measured under solar illumination is very good, with only one point of the fs-excited JV characteristic not perfectly overlapping the solar simulator excited one. Such small differences are likely due to differences in the spectral range of the femtosecond laser excitation source, for example. Nevertheless, it is worth noting that the open-circuit voltage is very similar in both measurements. This comparison, then, allows us to conclude that the carrier densities probed by the experiment are comparable to those of the operating solar cell. Working under such photoexcitation densities, Fig. 6 presents the 2DPCE spectra of the photovoltaic cell at a population waiting time of *t*_32_ = 0 fs. (see right-most, bottom inset of Fig. 6 for a definition of the inter-pulse delays). All spectra were measured keeping the cell under a bias voltage of ≈0.7 V. Shown are the real and imaginary parts of the rephasing (Fig. 6(a,b)), nonrephasing (Fig. 6(c,d)) spectra, and the total correlation function produced by the combination of these (Fig. 6(e,f)). As evident in Fig. 6(g), the laser spectrum covers the anticipated spectral range for the salient electronic states of this system, namely exciton and delocalised polarons, although it is narrow compared to the photocurrent excitation spectrum. In such conditions the bandwidth of the total correlation function is limited by the laser spectrum (see Fig. SI2). The total 2DPCE spectra in Fig. 6(e,f) reveal very little structure of the electronic states of the system. The main peak along the diagonal at 2.1 eV corresponds to the vertical exciton and one expects to see cross-peaks along the anti-diagonal corresponding to couplings to other electronic states coupled to the exciton. For the case at hand, the other electronic states of the system are optically dark or at best carry little oscillator strength to the ground state. As shown above by our quantum-dynamical modeling and Fig. 4, the fact that we see little trace of cross-peaks associated with these states but do see signal is indicative of very rapid electronic decoherence if resonance tunnelling from excitons to delocalised polarons brought about by bath-induced off-diagonal coupling as suggested in ref. 19 is the operative mechanism on these early time-scales. From these measurements and under the assumptions of our theoretical modelling, we conclude that the decoherence time between exciton and polaron photocarrier precursor states must be 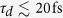. Moreover, no clear evolution of the 2DPCE spectra was observed for population times ranging between 0 fs and 180 fs (see Fig. SI 1).

**Figure 7:**
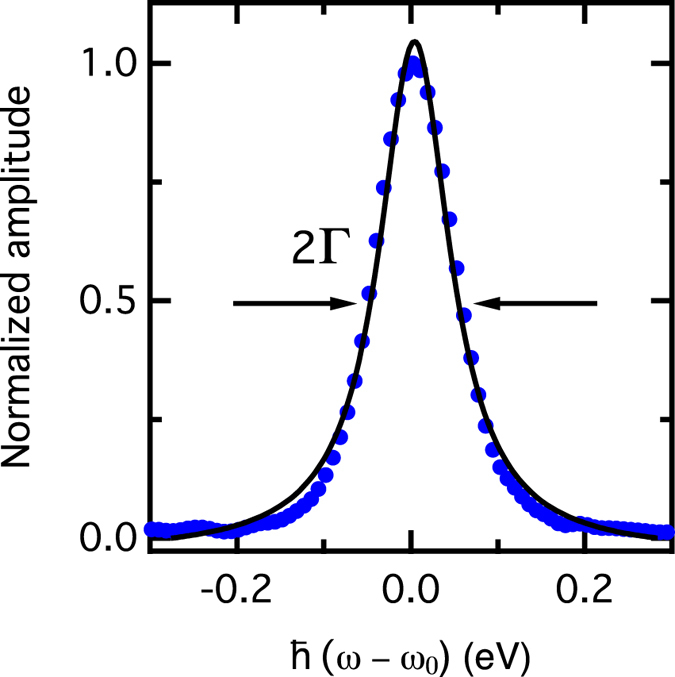
Anti-diagonal slice of the 2DPCE rephasing signal. The solid line is a fit to a Lorentzian function in which the optical dephasing rate Γ characterizes the homogeneous linewidth.

In order to explore the decoherence rate inferred above, we also seek to measure the optical dephasing time which can be measured from the anti-diagonal amplitude of the rephasing spectrum at population waiting time *t*_32_ = 0 fs (Fig. 6(a,b))^50^. Figure 7 shows such an antidiagonal cut, and the solid line is a fit to a Lorentzian function with full-width-at-half-maximum of 100 ± 1 meV, which represents the homogeneous linewidth of the main excitonic transition of PCDTBT over the spectral region covered by the ultrafast laser bandwidth. This implies a pure dephasing time 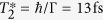, where 2Γ is the homogeneous linewidth. This dephasing time is consistent with the off-diagonal (exciton-polaron) decoherence time inferred above, which we rationalise with the fact that the two quantum dissipation processes are governed by the coupling of the system to the vibronic bath, and are subject to similar spectral density.

## Discussion

We present here a combined theoretical and experimental investigation into the origins of prompt photocarrier production in a working organic photovoltaic system. The correlation between the observed photocurrent with a sequence of ultrashort laser pulses supports our hypothesis that that off-diagonal couplings modulated by fluctuations within the bath enable resonant tunneling between an optically prepared exciton and a density of delocalized polaron states. In general any signal in the photocurrent is indicative of a direct tunneling process from exciton states close to the donor/acceptor interface to the continuum of CS states. In our experiment excitons produced far from the interface are removed from the signal by the pulse sequence and, even though they may give rise to a back-ground current, they would not contribute to the 4-th order correlation function, which the 2DPCE spectra are a measure of. Finally, it needs to be noted that all stray charges lingering from other processes and previous excitations are pushed out of the cell by the applied voltage bias.

In a recent paper Bässler and Köhler^51^ reviewed relevant experimental approaches and concepts proposed to describe the charge separation process in donor-acceptor organic solar cells, enforcing the notion that this occurs through a charge separated state that is a vibrationally cold charge transfer state. It is worth noting that our results are not at variance with those conclusions in that our experiments and theoretical modeling aimed to probe the sub-100 fs step of the charge photogeneration, whereas the dissociation process addressed by Bässler and Köhler occurs over time-scales spanning from 100 fs to ns.

The model and experimental results here reported corroborate and tie together a number of related recent investigations into the nature of the coupling between excitons and polarons in OPV systems^19^,^40^,^52^,^53^. In the present work the dephasing rate was introduced as a phenomenological parameter to compare the the experimental spectra computed ones. The dephasing rates can of course be related to energy gap fluctuations. In fact the phenomenological rates that produce the best match to the observations are consistent with the gap fluctuations Bittner and Kelley^52^ reported recently. Specifically, in ref. 52, mixed quantum/classical methods were used to sample the density of excited states produced by a polymer:fullerene contact pair undergoing molecular dynamics and it was found that on a very fast time-scale purely excitonic states rapidly mix with charge separated ones even after the initial excitation has thermalized with respect to the molecular motions. In ref. 40 the authors used *ab initio* quantum chemical methods to parametrize a fully quantum model of a polymer:fullerene heterojunction system. Quantum dynamics simulations on this system indicate that delocalization of charge in the fullerene phase reduces the Coulomb binding potential of the interfacial charge-transfer states and that vibrational excitations provide the necessary energy to facilitate charge separation. Along similar veins, efficient charge mobility can be attributed to the high degree of packing efficiency in the fullerene phase^53^. Here, resonant coupling of photogenerated singlet excitons to a high-energy manifold of fullerene electronic states enables efficient charge generation, bypassing localized charge-transfer states. While the theoretical approaches taken by each of these are very different, the underlying picture that emerges is excitons decay *directly* into states capable of producing photocurrent and that the time-scale for this process is ultimately governed by the spectral density of the environment that gives rise to energy level fluctuations. These same fluctuations also govern the optical dephasing time of the exciton and set the time-scale over which perturbative (*i*.*e*. Fermi golden-rule) descriptions of the decay are applicable.

Overall, the central results of the present work can be summarized as follows. Firstly, the detected photocurrent in the 2DPCE spectra is indicative of a direct tunneling process from an exciton state to any current-producing charge-separated state. Secondly, the absence of clear cross-peaks associated to other electronic state coupled to the exciton state and the absence of any evolution of the 2DPCE line shape for coherence times up to 200 fs can be interpreted, in the frame of our theoretical model, as evidence that the decoherence time between exciton and polaron photocarrier precursor states must be 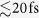. This is a negative result in the sense that we interpret lack of structure in the early-time 2DPCE spectrum as consistent with the ultrafast decoherence process. This observations is, nevertheless, significant in our view.

## Methods

### Solar cell preparation and characterization

The devices examined in the present work are based on blends of poly(N-90-heptadecanyl-2,7-carbazole-alt-5,5-(40,70- di-2-thienyl-20,10,30 -benzothiadiazole)) as electron donor and of the fullerene derivative [6,6]-phenyl-C60 butyric acid methyl ester as electron acceptor (PCDTBT:PCBM). The PV device structure is ITO/PEDOT:PSS/PCDTBT:PCBM/Ca/Al. PEDOT:PSS was spun onto a cleaned ITO coated glass substrate to form a film of 35 nm thickness. The active layer of thickness 80–90 nm was then spin cast on top of PEDOT:PSS layer from a blend of PCDTBT:PCBM (1:2) in chlorobenzene solvent with a concentration of 25 mg/ml. The top electrode calcium (≈20 nm) and aluminium (≈100 nm) was then subsequently deposited by thermal evaporation. Current density/voltage (J/V) characteristics of the devices were measured using a Keithley 236 Source Measure Unit. Solar cell performance was measured by using a xenon lamp with AM1.5 G filters and 100 mW/cm^−2^ illumination solar simulator (Oriel Instruments). In the photocurrent excitation measurement, the sample was illuminated at normal incidence by using a modulated laser light as an excitation source, the induced photocurrent was collected by a lock-in amplifier (SR830) referenced to the chopping frequency.

### 2DPCE Apparatus

**Figure 8:**
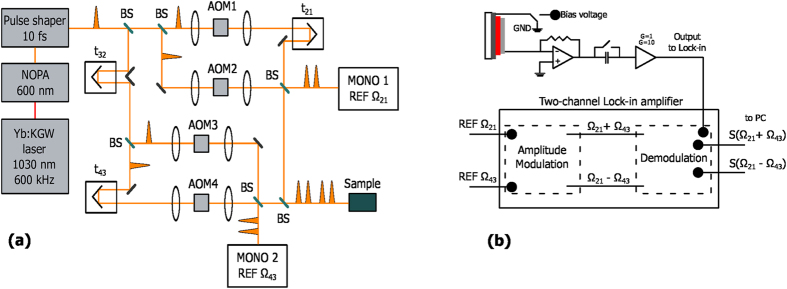
(**a**) Schematic diagram of the 2DPCE setup involving a Mach-Zehnder interferometer with inner Mach-Zehnder interferometers nested in each arm of the outer one. NOPA is noncollinear parametric amplifier, BS is beamsplitter, AOM is acousto-optic modulator, MONO is monochromator, *t*_*ij*_ is the temporal delay between pulse *i* and *j*, and REF is an optically generated reference signal waveform with frequency Ω_*ij*_. (**b**) Schematic diagram of the phase-sensitive detection setup involving amplitude modulation and lock-in amplification.

Our 2DPCE setup, shown in Fig. 8, is based on the phase-modulation and phase-sensitive detection system conceived by Marcus and co-workers^24^,^54^. It consists of a collinear four-pulse sequence (see bottom-right panel of Fig. 6) that is generated by means of two Mach-Zehnder interferometers nested in an outer Mach-Zehnder interferometer. The output of a non-collinear optical parametric amplifier (Light Conversion Orpheus-N-3H pumped by a Pharos regenerative amplifier operating at a repetition rate of 600 kHz) is pre-compressed by an adaptive 4*f* pulse shaper (Biophotonics Solution FemtoJock-P) in order to be <20 fs at the sample position. This output is split into two by means of a 50% beam splitter, and one arm is delayed with respect to the other by means of an optical delay time defining the delay variable *t*_32_, and then recombined into a collinear combination of the pulse trains by an identical beam splitter. Each of the two arms is then split into two sub-arms by identical 50% beam splitters and then recombined to generate the four-pulse sequence. The twin internal interferometers thus each generate two of the phase-locked pulses of the sequence. In order to ensure the phase locking condition, an acousto-optic Bragg cell is placed in each arm of the twin interferometers. The acousto-optic modulation imposes on each pulse of the sequence a frequency shift equal to the unique modulation frequency Ω_*i*_ (*i* = 1, 2, 3 and 4), close to 200 MHz. These frequency changes, small compared to the optical frequency, produce a shift in the temporal phase of each pulse which oscillates at the corresponding frequency Ω_*i*_. Each laser pulse is phase-shifted with respect to the previous shot, thus giving rise to two collinear trains of phase-modulated pulse pairs. When incident on a photodetector, the interfering excitation pulses produce a population signal oscillating at Ω_21_ = Ω_2_ − Ω_1_ and Ω_43_ = Ω_4_ − Ω_3_. The exact Ω_*i*_ values are chosen so that Ω_21_ and Ω_43_ are in the kHz range (at 5 and 8 kHz respectively, in the present case). This phase modulation scheme allows one to isolate the fourth-order contribution to the photocurrent signal by means of a lock-in amplifier (Zurich Instruments HF2LI equipped with multi-frequency and AM/FM modulation modules), which is then used to construct the 2D maps. Reference waveforms of frequency Ω_43_ − Ω_21_ and Ω_43_ + Ω_21_ are thus generated by amplitude modulation, and are used to demodulate the output of the sample solar cell. A Zurich Instruments HF2TA Current Amplifier is used to convert the current output of the sample device to voltage, as well as to supply an external bias to the device. The non-linear signals of interest oscillate at the frequencies Ω_43_ − Ω_21_ and Ω_43_ + Ω_21_ and in 2D spectroscopy these are usually referred to respectively as *rephasing* and *non-rephasing* signals. The overall photocurrent signal is filtered by means of a dual lock-in amplifier in order to extract these two frequency components. The 2D spectra are obtained measuring the demodulated signals scanning *t*_21_ and *t*_43_ at fixed *t*_32_. The three interpulse delay times *t*_21_, *t*_32_ and *t*_43_ are independently computer controlled using three delay stages. The in-phase and the in-quadrature detection channels of the dual lock-in amplifier simultaneously provide the real and imaginary parts of the rephasing and non-rephasing signals for each of such scans. The 2D response function so acquired in the time domain is converted to the energy domain by Fourier-transforming the time variables *t*_21_ and *t*_43_ as a parametric function of the *t*_32_ interpulse time.

## Additional Information

**How to cite this article**: Vella, E. *et al*. Ultrafast decoherence dynamics govern photocarrier generation efficiencies in polymer solarcells. *Sci. Rep*. **6**, 29437; doi: 10.1038/srep29437 (2016).

## Supplementary Material

Supplementary Information

## References

[b1] Bredas J.-L. When electrons leave holes in organic solar cells. Science 343, 492–493 (2014). 24482470 10.1126/science.1249230

[b2] Gélinas S. . The Binding Energy of Charge-Transfer Excitons Localized at Polymeric Semiconductor Heterojunctions. J. Phys. Chem. C 115, 7114–7119 (2011).

[b3] Sariciftci N. S. & Heeger A. J. Reversible, metastable, ultrafast photoinduced electron-transfer from semiconducting polymers to buckminsterfullerene and in the corresponding donor-acceptor heterojunctions. International Journal of Modern Physics B 8, 237–274 (1994).

[b4] Banerji N., Cowan S., Leclerc M., Vauthey E. & Heeger A. J. Exciton Formation, Relaxation, and Decay in PCDTBT. Journal of the American Chemical Society 132, 17459–17470 (2010). 21087001 10.1021/ja105290e

[b5] Tong M. . Charge carrier photogeneration and decay dynamics in the poly(2,7-carbazole) copolymer PCDTBT and in bulk heterojunction composites with PC_70_BM. Phys. Rev. B 81, 125210 (2010).

[b6] Sheng C.-X., Basel T., Pandit B. & Vardeny Z. V. Photoexcitation dynamics in polythiophene/fullerene blends for photovoltaic applications. Organic Electronics 13, 1031–1037 (2012).

[b7] Jailaubekov A. E. . Hot charge-transfer excitons set the time limit for charge separation at donor/acceptor interfaces in organic photovoltaics. Nat Mater 12, 66–73 (2013). 23223125 10.1038/nmat3500

[b8] Grancini G. . Hot exciton dissociation in polymer solar cells. Nat Mater 12, 29–33 (2013). 23223127 10.1038/nmat3502

[b9] Kaake L. G., Moses D. & Heeger A. J. Coherence and uncertainty in nanostructured organic photovoltaics. The Journal of Physical Chemistry Letters 4, 2264–2268 (2013). 10.1021/jp408700m 24070027

[b10] Mukamel S. Comment on “Coherence and uncertainty in nanostructured organic photovoltaics”. The Journal of Physical Chemistry A 117, 10563–10564 (2013). 24032455 10.1021/jp4071086

[b11] Banerji N. Sub-picosecond delocalization in the excited state of conjugated homopolymers and donor–acceptor copolymers. J. Mater. Chem. C 1, 3052–3066 (2013).

[b12] Gélinas S. . Coherent charge separation in organic semiconductor photovoltaic diodes. Science 343, 521–516 (2014). 10.1126/science.1246249 24336568

[b13] Provencher F. . Direct observation of ultrafast long-range charge separation at polymer:fullerene heterojunctions. Nat Commun 5, 4288 (2014). 24980429 10.1038/ncomms5288

[b14] Barbara P., Meyer T. & Ratner M. Contemporary issues in electron transfer research. J Phys Chem-Us 100, 13148–13168 (1996).

[b15] Falke S. M. . Coherent ultrafast charge transfer in an organic photovoltaic blend. Science 344, 1001–1005 (2014). 24876491 10.1126/science.1249771

[b16] Ishizaki A. & Fleming G. R. Theoretical examination of quantum coherence in a photosynthetic system at physiological temperature. Proceedings of the National Academy of Sciences 106, 17255–17260 (2009). 10.1073/pnas.0908989106 PMC2762676 19815512

[b17] Song Y., Clafton S. N., Pensack R. D., Kee T. W. & Scholes G. D. Vibrational coherence probes the mechanism of ultrafast electron transfer in polymer&ndash;fullerene blends. Nature Communications 5, 4933 (2014). 10.1038/ncomms5933 25215959

[b18] Andrea Rozzi C. . Quantum coherence controls the charge separation in a prototypical artificial light-harvesting system. Nat Commun 4, 1602 (2013). 23511467 10.1038/ncomms2603 PMC3615481

[b19] Bittner E. R. & Silva C. Noise-induced quantum coherence drives photo-carrier generation dynamics at polymeric semiconductor heterojunctions. Nat Commun 5, 3119 (2014). 24477069 10.1038/ncomms4119

[b20] Yao Y., Zhou N., Prior J. & Zhao Y. Competition between diagonal and off-diagonal coupling gives rise to charge-transfer states in polymeric solar cells. Scientific Reports 5, 14555 (2015). 26412693 10.1038/srep14555 PMC4585960

[b21] Zhao Y., Yao Y., Chernyak V. & Zhao Y. Communication: Spin-boson model with diagonal and off-diagonal coupling to two independent baths: Ground-state phase transition in the deep sub-ohmic regime. J Chem Phys 140, 161105 (2014). 24784244 10.1063/1.4873351

[b22] Bakulin A. A., Silva C. & Vella E. Ultrafast spectroscopy with photocurrent detection: Watching excitonic optoelectronic systems at work. J. Phys. Chem. Lett. 7, 250–258 (2016). 26711855 10.1021/acs.jpclett.5b01955 PMC4819534

[b23] Nardin G., Autry T. M., Silverman K. L. & Cundiff S. T. Multidimensional coherent photocurrent spectroscopy of a semiconductor nanostructure. Opt. Express 21, 28617–28627 (2013). 24514373 10.1364/OE.21.028617

[b24] Karki K. J. . Coherent two-dimensional photocurrent spectroscopy in a PbS quantum dot photocell. Nat. Commun. 5, 5869 (2014). 25519819 10.1038/ncomms6869

[b25] Few S., Frost J. M. & Nelson J. Models of charge pair generation in organic solar cells. Phys. Chem. Chem. Phys. 17, 2311–2325 (2015). 25462189 10.1039/c4cp03663h

[b26] Few S., Frost J. M., Kirkpatrick J. & Nelson J. Influence of chemical structure on the charge transfer state spectrum of a polymer:fullerene complex. Journal of Physical Chemistry C 118, 8253–8261 (2014).

[b27] Goris L. . Absorption phenomena in organic thin films for solar cell applications investigated by photothermal deflection spectroscopy. Journal of Materials Science 40, 1413–1418 (2005).

[b28] Beenken W. J. D. . Sub-bandgap absorption in organic solar cells: experiment and theory. Phys. Chem. Chem. Phys. 15, 16494–16502 (2013). 23929440 10.1039/c3cp42236d

[b29] Goris L. . Observation of the subgap optical absorption in polymer-fullerene blend solar cells. Applied Physics Letters 88, 052113 (2006).

[b30] Benson-Smith J. . Formation of a ground-state charge-transfer complex in polyfluorene//[6,6]-phenyl-C61 butyric acid methyl ester (PCBM) blend films and its role in the function of polymer/PCBM solar cells. Advanced Functional Materials 17, 451–457 (2007).

[b31] Loi M. . Charge transfer excitons in bulk heterojunctions of a polyfluorene copolymer and a fullerene derivative. Advanced Functional Materials 17, 2111–2116 (2007).

[b32] Tvingstedt K. . Electroluminescence from charge transfer states in polymer solar cells. Journal of the American Chemical Society 131, 11819–11824 (2009). 19722595 10.1021/ja903100p

[b33] Niedzialek D. . First principles calculations of charge transfer excitations in polymer–fullerene complexes: Influence of excess energy. Advanced Functional Materials 25, 1972–1984 (2015).

[b34] Isaacs E. B., Sharifzadeh S., Ma B. & Neaton J. B. Relating trends in first-principles electronic structure and open-circuit voltage in organic photovoltaics. The Journal of Physical Chemistry Letters 2, 2531–2537 (2011).

[b35] Baumeier B., Andrienko D. & Rohlfing M. Frenkel and charge-transfer excitations in donor–acceptor complexes from many-body green’s functions theory. Journal of Chemical Theory and Computation 8, 2790–2795 (2012). 26592120 10.1021/ct300311x

[b36] Yost S. R. & Voorhis T. V. Electrostatic effects at organic semiconductor interfaces: A mechanism for “cold” exciton breakup. The Journal of Physical Chemistry C 117, 5617–5625 (2013).

[b37] Poelking C. . Impact of mesoscale order on open-circuit voltage in organic solar cells. Nat Mater 14, 434–439 (2015). 25532071 10.1038/nmat4167

[b38] Karabunarliev S. & Bittner E. R. Spin-dependent electron-hole capture kinetics in luminescent conjugated polymers. Physical Review Letters 90, 057402 (2003). 12633395 10.1103/PhysRevLett.90.057402

[b39] Karabunarliev S. & Bittner E. R. Polaron–excitons and electron–vibrational band shapes in conjugated polymers. The Journal of Chemical Physics 118, 4291–4296 (2003).

[b40] Savoie B. M. . Unequal partnership: Asymmetric roles of polymeric donor and fullerene acceptor in generating free charge. Journal of the American Chemical Society 136, 2876–2884 (2014). 24460057 10.1021/ja411859m

[b41] Raos G., Casalegno M. & Idé J. An effective two-orbital quantum chemical model for organic photovoltaic materials. Journal of Chemical Theory and Computation 10, 364–372 (2014). 26579915 10.1021/ct400854a

[b42] Bakulin A. A. . The role of driving energy and delocalized states for charge separation in organic semiconductors. Science 335, 1340–1344 (2012). 22362882 10.1126/science.1217745

[b43] Gutiérrez-González I., Molina-Brito B., Götz A. W., Castillo-Alvarado F. & Rodríguez J. I. Structural and electronic properties of the p3ht–pcbm dimer: A theoretical study. Chemical Physics Letters 612, 234–239 (2014).

[b44] Liu Y., Chipot C., Shao X. & Cai W. Free-energy landscape of the helical wrapping of a carbon nanotube by a polysaccharide. The Journal of Physical Chemistry C 115, 1851–1856 (2011).

[b45] Alicki R. & Lendi K. Quantum Dynamical Semigroups and Applications (Springer, Berlin, 1987).

[b46] Kosloff R., Ratner M. A. & Davis W. B. Dynamics and relaxation in interacting systems: Semigroup methods. J. Chem. Phys. 106, 7036–7043 (1997).

[b47] Perdomo-Ortiz A., Widom J. R., Lott G. A., Aspuru-Guzik A. & Marcus A. H. Conformation and electronic population transfer in membrane-supported self-assembled porphyrin dimers by 2d fluorescence spectroscopy. J. Phys. Chem. B 116, 10757–10770 (2012). 22882118 10.1021/jp305916x

[b48] Park S. H. . Bulk heterojunction solar cells with internal quantum efficiency approaching 100%. Nat. Photonics 3, 297–302 (2009).

[b49] Sun Y. . Efficient, Air-Stable Bulk Heterojunction Polymer Solar Cells Using MoOx as the Anode Interfacial Layer. Advanced Materials 23, 2226–2230 (2011). 21469222 10.1002/adma.201100038

[b50] Tokmakoff A. Two-Dimensional Line Shapes Derived from Coherent Third-Order Nonlinear Spectroscopy. J. Phys. Chem. A 104, 4247–4255 (2000).

[b51] Bässler H. & Köhler A. “Hot or cold”: how do charge transfer states at the donor-acceptor interface of an organic solar cell dissociate? Phys. Chem. Chem. Phys. 17, 28451–28462 (2015). 26456722 10.1039/c5cp04110d

[b52] Bittner E. R. & Kelley A. The role of structural fluctuations and environmental noise in the electron/hole separation kinetics at organic polymer bulk-heterojunction interfaces. Phys. Chem. Chem. Phys. 17, 28853–28859 (2015). 26449151 10.1039/c5cp05037e

[b53] Huix-Rotllant M., Tamura H. & Burghardt I. Concurrent effects of delocalization and internal conversion tune charge separation at regioregular polythiophene–fullerene heterojunctions. The Journal of Physical Chemistry Letters 6, 1702–1708 (2015). 26263337 10.1021/acs.jpclett.5b00336

[b54] Tekavec P. F., Lott G. A. & Marcus A. H. Fluorescence-detected two-dimensional electronic coherence spectroscopy by acousto-optic phase modulation. J. Chem. Phys. 127, 214307 (2007). 18067357 10.1063/1.2800560

