# Supplementary material for: Ultrafast decoherence dynamics govern photocarrier generation efficiencies in polymer solar cells
Source: Sci Rep. 2016 Jul 14;6:29437. doi: 10.1038/srep29437 (PMC4944175; doi:10.1038/srep29437)
Supplement: Supplementary Information [file srep29437-s1.pdf]

Supplementary Information: Ultrafast decoherence dynamics govern photocarrier generation efficiencies in polymer solar cells

Eleonora Vella, Pascal Grégoire, Claudia M. Bazan, and Carlos Silva

Département de physique & Regroupement québécois sur les matériaux de pointe,

Université de Montréal, C.P. 6128, Succursale centre-ville,

Montréal (Québec) H3C 3J7, Canada

Hao Li and Eric R. Bittner

Departments of Chemistry and Physics,

University of Houston, Houston, Texas 77204, USA

Sachetan M. Tuladhar, Michelle Vezie, Sheridan Few and Jenny Nelson

Department of Physics, Imperial College London,

South Kensington Campus, London SW7 2AZ, United Kingdom

I. TOTAL CORRELATION FUNCTION AND LASER SPECTRUM BANDWIDTH

The photocurrent excitation (PCE) spectrum is broader than the laser spectrum, as results evident from Fig. 6(g) of the main text. In such a condition, the TCF signal is expected to have a similar bandwidth as the laser spectrum. To show that this is indeed the case, in figure 1 (a) the norm of the TCF signal at t_{32}=0 fs is reported on a normalized scale. Moreover, in Figure 1 (b) a cut along the diagonal of the 2D spectrum in (a) is shown together with the laser spectrum: as one can see these two profiles have a comparable bandwidth. In Figure 6 of the main text with the aim of having a uniform scale for the real and imaginary part of each signal (rephasing, non-rephasing and total correlation function), the color scale is more expanded and the TCF thus appears narrower than the laser spectrum, when instead the two have a similar bandwidth. In our experimental condition, then, the TCF signal is limited by the bandwidth of the laser. It is worth noting, though, that the spectrum of the laser has a bandwidth of ~110 meV. This means that the bandwidth of the spectrum of the laser is, indeed, wide enough to detect structures associated to a coherent coupling between the exciton and the charge separated states, if there was any to be seen within the given dephasing time.

II. T_{32} TIME EVOLUTION

The rephasing and non-rephasing pathways contain diagrams involving excited state population evolution during t_{32}. In order to measure the population decay time and to possibly compare it to the optical dephasing time to see if it is the dominant contribution we measure the TCF spectra at different t_{32} times. These are reported in figure 2. Fig.2 specifically shows the real and imaginary parts of the TCF signal at t_{32}=0 fs (a and b respectively), at t_{32}=50 fs (c and d respectively) and at t_{32}=180 fs (e and f respectively). As one can see, no clear evolution of the signals can be detected over this time range. The absence of a t_{32} time evolution of the 2D spectra is a consequence of the fact that the measured TCF signal is limited by the bandwidth of the laser.

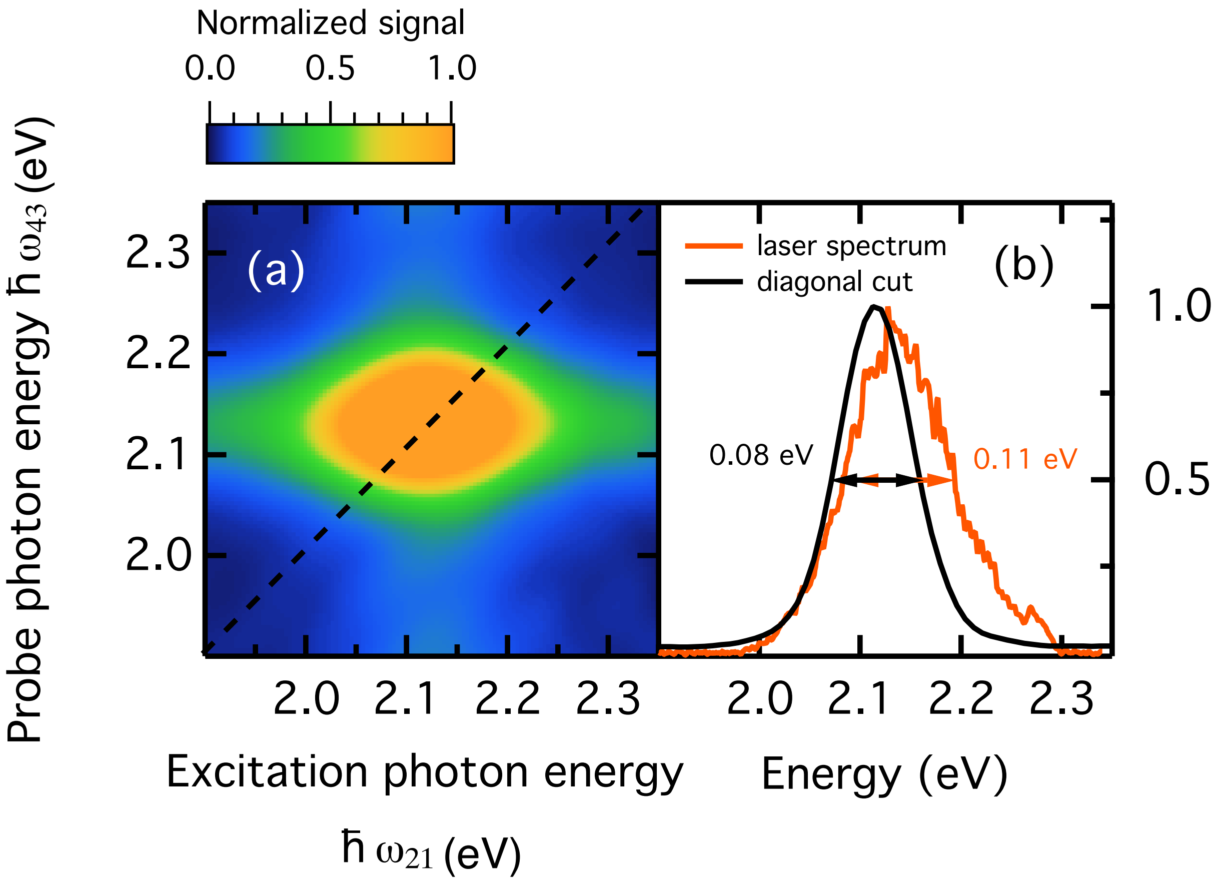

FIG. 1. (a) Norm of the total correlation function (TCF) at t_{32}=0 fs. (b) Diagonal cut of the 2D spectrum in (a) and normalized laser excitation spectrum. The FWHM for the two profiles are also reported.

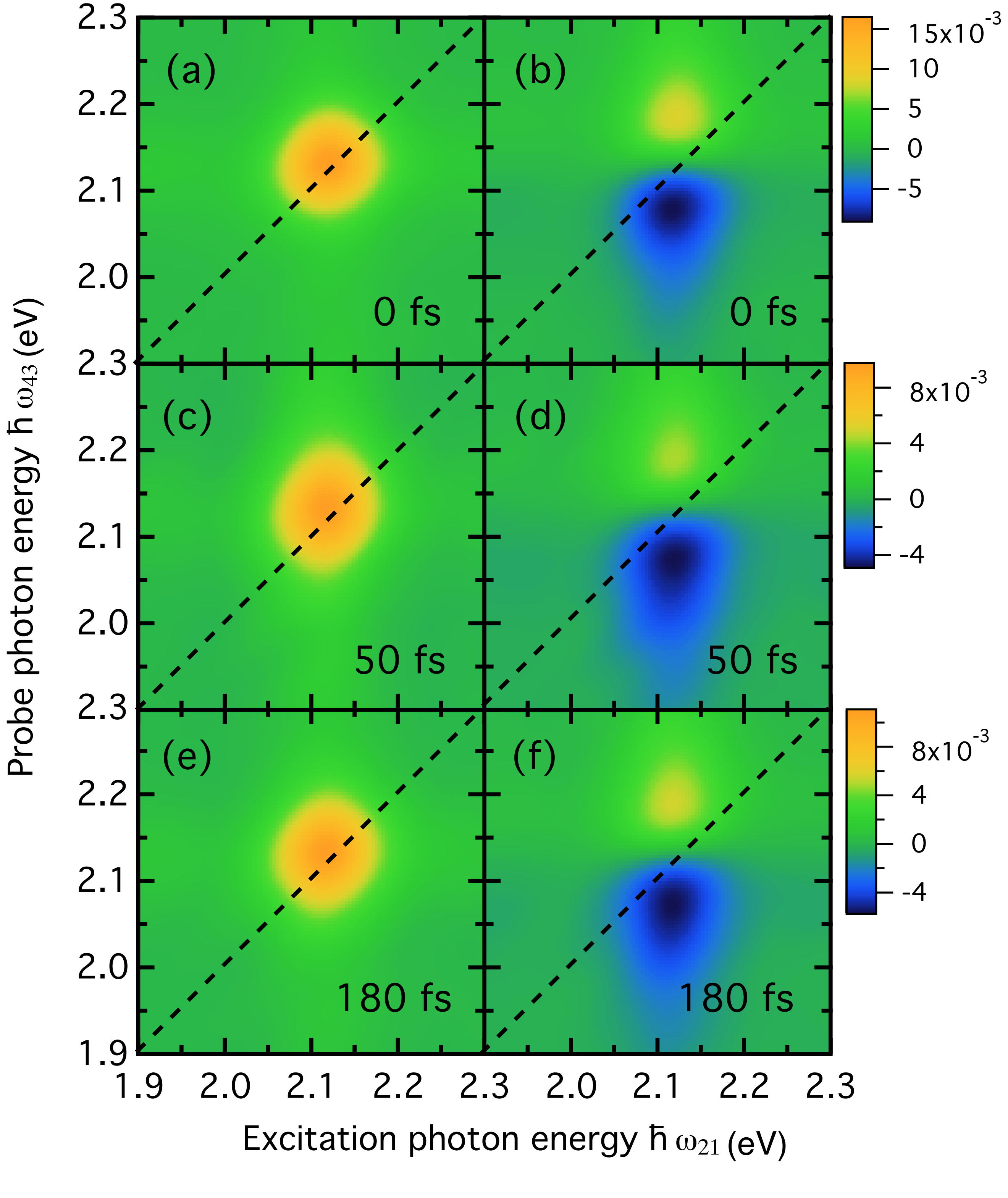

FIG. 2. TCF spectra at different t_{32} times: t_{32}=0 fs, real and imaginary parts (a)-(b); t_{32}=50 fs, real and imaginary parts (c)-(d) and t_{32}=180 fs, real and imaginary parts (e)-(f).
